# *Exophiala (Wangiella) dermatitidis* Prosthetic Aortic Valve Endocarditis and Prosthetic Graft Infection in an Immune Competent Patient

**DOI:** 10.1155/2017/4839314

**Published:** 2017-04-18

**Authors:** Jay S. Berger, Lucas R. Cusumano, Joseph J. Derose, Uzma N. Sarwar

**Affiliations:** ^1^Department of Anesthesiology, Division of Critical Care Medicine, Albert Einstein College of Medicine, Montefiore Medical Center, Bronx, NY, USA; ^2^Albert Einstein College of Medicine, Montefiore Medical Center, Bronx, NY, USA; ^3^Department of Cardiovascular and Thoracic Surgery, Albert Einstein College of Medicine, Montefiore Medical Center, Bronx, NY, USA; ^4^Division of Infectious Diseases, Albert Einstein College of Medicine, Montefiore Medical Center, Bronx, NY, USA

## Abstract

*Exophiala (Wangiella) dermatitidis* is an emerging dematiaceous fungus associated with high mortality rates and is a rare cause of endocarditis. We describe the first case of* E. dermatitidis* endocarditis of a prosthetic aortic valve and aortic graft in an immune competent patient with no clear risk factors of hematological acquisition.

## 1. Introduction

*Exophiala (Wangiella) dermatitidis* is a dematiaceous fungus that has been isolated from soil, decaying organic matter, plant debris, and human feces. Recent reports have also described isolation in indoor environments such as kitchen sinks and dishwashers and in steam baths and tubs [1–5]. Infections caused by* E. dermatitidis* are called phaeohyphomycosis due to the presence of dark pigmented hyphal elements that are seen on histopathology [3]. Phaeohyphomycosis may involve any organ of the body and infections involving the skin, brain, lung, eye, joints, and endocardium have been previously reported [3, 6]. However, the route of human systemic infection remains elusive. Infections with this fungus are rare but tend to be associated with a high mortality rate [7]. To our knowledge, there have been only two previous cases reported of aortic valve endocarditis secondary to* E. dermatitidis* infection. One occurred in a patient who was immune compromised and the second case was in a patient with a clear risk of acquisition in the setting of active intravenous drug use [8, 9]. The case we discuss below is the first report of* E. dermatitidis *infection of a prosthetic aortic valve and aortic graft in an immune competent patient.

## 2. Case

A 53-year-old African American male presented to the Weiler Campus of Montefiore Medical Center on January 12, 2016, with a two-week history of intermittent fevers and chest pain. His past medical history was significant for hypertension and an emergency repair of a ruptured aortic root aneurysm with a valve-sparing aortic root replacement in August 2014. His postoperative course at this time was unremarkable. Four days prior to his current admission, he was seen by his primary care physician with complaints of fever and cough. He received a course of oral antibiotics for “walking pneumonia.” His symptoms did not improve and he presented to the hospital after a syncopal episode at home.

He was born in New York with no relevant travel history. He was a former interior decorator and routinely did woodwork but stopped working after his surgery in 2014. He lived at home with his wife and eight-year-old daughter. They have one cat and no other pets. He occasionally smokes marijuana.

On presentation to the Emergency Department he was hypotensive and hypoxic, but afebrile. His initial white blood cell (WBC) count was 19 and his hemoglobin was 8.2 mg/dl. Computed tomography angiography (CTA) of his chest showed fluid around the aortic root without extravasation. A transesophageal echocardiogram revealed multiple small mobile densities (3-4 mm) on the aortic valve and inner surface of the aortic graft with moderate aortic valve regurgitation. He also had circumferential thickening outside the aortic graft suggestive of a paragraft abscess. He was started on broad spectrum antibiotics (vancomycin 1 g, 12 hourly and piperacillin-tazobactam 4.5 g every 6 hours) and multiple sets of blood cultures were sent which remained negative.

On hospital day 7, he underwent a reoperative mechanical aortic valve and partial aortic arch replacement with reimplantation of the innominate artery. Intraoperative findings were significant for a periaortic abscess. A gram stain of the graft showed many polymorphonuclear leukocytes. Cytopathology revealed marked acute and chronic inflammation of the aortic valve consistent with acute endocarditis as well as focal necrosis and exudate of the aortic graft consistent with an abscess.

**Figure 1:**
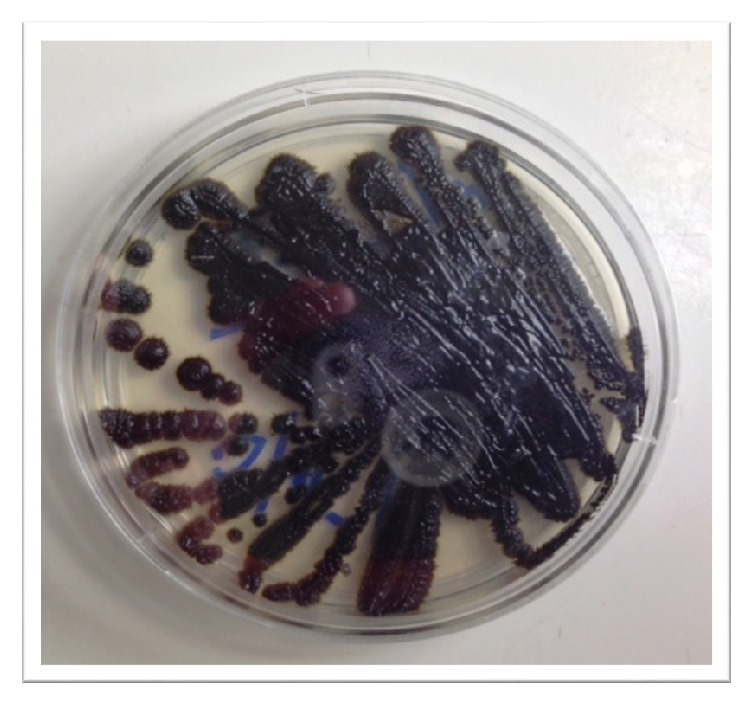
*Exophiala dermatitidis* growing on Sabouraud Dextrose agar at 37 degrees Celsius.

Culture of the aortic valve initially on thioglycolate broth showed budding yeast. Subculture of the isolate on Sabouraud Dextrose agar grown at 37 degrees Celsius yielded black colonies of yeast (Figure 1). On observing the colonies with Lactophenol cotton blue by doing tape preparation, dark, septate, and cylindrical to flask shaped conidiogenous cells were seen. Round to oval single celled pale brown conidia accumulate at the apex of the conidiogenous cell and down the sides of the conidiophore were observed. The isolate from the aortic valve culture was identified as* E. dermatitidis* by the microbiology laboratory at our institution using the BD Phoenix automated microbiology system (Becton Dickinson Diagnostic Systems Phoenix Instrument, Maryland, United States) [10].

Further molecular characterization and confirmation of the pathogenic fungal organism as* Exophiala dermatitidis* was performed by the Mycology Laboratory at the New York State Department of Health (NYSDOH). In addition to phenotypic characterization, internal transcribed spacer (ITS) sequencing was completed. The genomic DNA from the mold culture was isolated and the ITS region (ITS1-5.8S-ITS2) of the ribosomal gene was amplified using primer set V1827 (ITS5) 5′-GGAAGTAAAAGTCGTAACAAGG-3′ and V50 (ITS4) 5′-TCCTCCGCTTATTGATATGC-3′. Polymerase chain reaction (PCR) was performed as described previously [11]. Sequencing and BLAST search using two databases, GenBank (https://www.ncbi.nlm.nih.gov/genbank/) and Centraalbureau voor Schimmelcultures (http://www.fungalbarcoding.org/BioloMICSSequences.aspx), identified the PCR amplicon to be 100% identical to* E. dermatitidis*.

Gömöri methenamine silver (GMS) staining was performed on the histological specimens available and was negative for fungal organisms. In addition, a bacterial gram stain and acid-fast bacilli (AFB) stain were also negative. On hospital day 16, Micafungin 100 mg daily was added to his antimicrobial regimen once yeast was isolated from his operative cultures. After the identification of the yeast as* E. dermatitidis*, antifungal therapy was switched to intravenous Voriconazole (loading dose of 6 mg/kg twice daily; maintenance dose 4 mg/kg twice daily) and later converted to oral. The patient's postoperative course was marked by three separate reoperations for bleeding and tamponade (hospital days 10, 20, and 32), each time in the setting of an INR between 2.0 and 2.5. His postoperative course was further complicated by acute kidney injury, which later improved. A six-week course of empiric antibiotics was completed for possible bacterial endocarditis. He remained on oral Voriconazole until discharge from the hospital on day 63 with plans to continue as long as tolerated. He was kept on low dose Warfarin with an INR goal of 1.5–2.0. Extensive workup to evaluate an underlying immunodeficiency was unremarkable.

The in vitro antifungal susceptibility of our isolate of* E. dermatitidis* was determined using the broth microdilution method according to the guidelines of Clinical Laboratory Standards Institute (CLSI) at the NYSDOH. The minimum inhibitory concentrations (MIC) of Amphotericin B, Posaconazole, Voriconazole, Itraconazole, Fluconazole, Caspofungin, and Micafungin against the culture isolate were 0.5 mcg/mL, 0.06 mcg/mL, 0.25 mcg/mL, 4.0 mcg/mL, 4.0 mcg/mL, and 2.0 mcg/mL, respectively. The interpretations for the drugs were based on the CLSI M38-A2 document [12].

## 3. Discussion

*E. dermatitidis* rarely causes clinical infection in immune competent individuals. Clinical manifestations of disease can range from localized subcutaneous nodules to highly invasive infections such as brain abscesses, meningitis, and endocarditis [1, 6, 8]. Fungi are a less common cause of infective endocarditis and account for less than 10 percent of cases [13]. There are only two previous reports of* E. dermatitidis* endocarditis in the literature [8, 9]. As described earlier, Vartian et al. reported the first known case of endocarditis secondary to* E. dermatitidis* with a clear mode of acquisition in the setting of intravenous drug use. This patient's endocarditis affected the native aortic valve and was complicated by relapsing infection of subsequent aortic prosthesis and dissemination to the spine [9]. Patel et al. reported a case of native aortic valve endocarditis in a posttransplant patient on immune suppression with prednisolone, tacrolimus, and mycophenolate, who responded well to medical and surgical management [8]. Our case is the first report of endocarditis and prosthetic graft infection in a patient with no known underlying immunodeficiency and no clear risk factors of hematogenous acquisition such as intravenous drug use.

Diagnosis of fungal endocarditis can be challenging as the most common presenting clinical features such as fever, new heart murmur, and peripheral embolization are nonspecific for fungal etiologies [13, 14]. Even when there is a high index of suspicion, microbiological identification of fungi remains cumbersome. Identification of phaeohyphomycosis is particularly difficult as cultures for* E. dermatitidis* may remain negative even when infection is present [14]. This organism grows slowly on primary isolation media and recovery may be missed if blood cultures are incubated for short periods of time.

In this case, budding yeasts were seen from the patient's operative cultures after nine days of incubation. However, multiple sets of blood cultures remained negative despite an extended period of incubation of four weeks to ensure recovery of* E. dermatitidis*. Given his CTA, echocardiogram, and cytopathology findings, we considered the positive intraoperative prosthetic graft culture as evidence of infection with* E. dermatitidis* even though it was not identified from the patient's blood.

Extensive workup was completed to evaluate underlying immunodeficiency in our patient which was all unremarkable. Screening tests for human immunodeficiency virus (HIV) infection, immunoglobulin levels, lymphocyte subsets, and peripheral flow cytometry were all within normal limits. In addition, serological markers of connective tissue disease were negative as well. No underlying immunologic predisposition was identified and we presumed that our patient acquired this pathogen via inhalational exposure leading to hematogenous spread. However, our patient did have two of the most common risk factors for fungal endocarditis: previous cardiac surgery for an aortic root aneurysm and recent antibiotic treatment for pneumonia [13, 14].

Perhaps even more challenging than accurate diagnosis is the appropriate clinical management of this infection. There are no previous randomized controlled trials addressing treatment guidelines for* E. dermatitidis *and all present data in current literature is based on case reports and series. Treatment is dependent on the site of infection and can range from surgical excision alone for localized subcutaneous infection to a multipronged approach with surgical debridement combination antifungal therapy as well as immune enhancement for cerebral phaeohyphomycosis [3, 7, 8, 13, 15].

With involvement of the prosthetic aortic valve and graft, our patient underwent aortic valve replacement with a bioprosthetic valve [3, 16]. Voriconazole was chosen because of favorable treatment responses in previous reports of* E. dermatitidis* endocarditis, tolerability, and the isolate's susceptibility profile [8]. Furthermore, due to postoperative complications of acute kidney injury we decided to avoid Amphotericin B because of its known risk of nephrotoxicity.

Another important issue with* E. dermatitidis* invasive infections is to define an ideal duration of treatment. We plan to continue an extended course of oral Voriconazole with close clinical monitoring given the severity of his infection and the involvement of prosthetic materials. At this juncture it is hard to define a finite duration of treatment weighing the risks of reinfection of the new valve and graft with the side effects of treatment. With the lack of clinical trials data and no established treatment guidelines, further investigation is needed to define the long-term management of* E. dermatitidis* infections.
